# A Novel Splice-Site Mutation in the *GJB2* Gene Causing Mild Postlingual Hearing Impairment

**DOI:** 10.1371/journal.pone.0073566

**Published:** 2013-09-06

**Authors:** Marta Gandía, Francisco J. del Castillo, Francisco J. Rodríguez-Álvarez, Gema Garrido, Manuela Villamar, Manuela Calderón, Miguel A. Moreno-Pelayo, Felipe Moreno, Ignacio del Castillo

**Affiliations:** 1 Unidad de Genética Molecular, Hospital Universitario Ramón y Cajal, Instituto Ramón y Cajal de Investigación Sanitaria (IRYCIS), Madrid, Spain; 2 Centro de Investigación Biomédica en Red de Enfermedades Raras (CIBERER), Madrid, Spain; 3 Servicio de Otorrinolaringología, Hospital Universitario Ramón y Cajal, Madrid, Spain; Innsbruck Medical University, Austria

## Abstract

The DFNB1 subtype of autosomal recessive, nonsyndromic hearing impairment, caused by mutations affecting the *GJB2* (connection-26) gene, is highly prevalent in most populations worldwide. DFNB1 hearing impairment is mostly severe or profound and usually appears before the acquisition of speech (prelingual onset), though a small number of hypomorphic missense mutations result in mild or moderate deafness of postlingual onset. We identified a novel *GJB2* splice-site mutation, c. -22-2A>C, in three siblings with mild postlingual hearing impairment that were compound heterozygous for c. -22-2A>C and c.35delG. Reverse transcriptase-PCR experiments performed on total RNA extracted from saliva samples from one of these siblings confirmed that c. -22-2A>C abolished the acceptor splice site of the single *GJB2* intron, resulting in the absence of normally processed transcripts from this allele. However, we did isolate transcripts from the c. -22-2A>C allele that keep an intact *GJB2* coding region and that were generated by use of an alternative acceptor splice site previously unknown. The residual expression of wild-type connection-26 encoded by these transcripts probably underlies the mild severity and late onset of the hearing impairment of these subjects.

## Introduction

Hereditary non-syndromic hearing impairment (NSHI) is a heterogeneous genetic condition. To date, mutations in 73 different genes have been shown to cause NSHI. At least as many genes remain to be identified, as indicated by the number of known NSHI loci for which the underlying causative mutations have not been found yet (http://hereditaryhearingloss.org/) [1]. This high genetic heterogeneity mirrors the structural and functional complexity of the hearing process and it is a major hurdle for the successful genetic diagnosis of subjects with NSHI.

The DFNB1 subtype of autosomal recessive NSHI is remarkable for being highly prevalent in most of the populations tested so far (reviewed in 2). The underlying locus, DFNB1, lies on 13q12 and encompasses the *GJB2* gene (MIM # 121011), which encodes the gap-junction protein connection-26 (Cx26). *GJB2* consists of a 160-bp non-coding exon, a single 3,179-bp intron and a 2,134-bp exon that contains 22 bp of the 5’-untranslated region (UTR), the complete 678-bp coding sequence and the 3’-UTR (UniGene Hs.524894) [3].

Mutations at the DFNB1 locus can be classified in two groups: (i) those that affect the coding sequence of *GJB2*; and (ii) those that lie outside the coding sequence of *GJB2* and affect the expression and/or regulation of this gene [2]. Since screening of *GJB2* is considered the gold standard of genetic diagnosis of hereditary HI, it is not surprising that more than 100 pathogenic mutations in the *GJB2* coding sequence have been identified so far (http://davinci.crg.es/deafness/) [4]. A few *GJB2* mutations predominate in particular populations due to demonstrated founder effects [5–11].

By contrast, only six pathogenic mutations are known outside the *GJB2* coding sequence. Two of them are point mutations: c. -23+1G > A (originally named IVS1+1G>A), the only mutation known to affect the donor splice site of the single intron [12], and g. -77C>T (originally named -3438C>T), which abolishes the activity of the basal promoter of the gene [13]. The remaining four mutations are large deletions. One of them is a deletion of about 920 kb that encompasses the complete *GJB2* gene [14], whereas the three other deletions (del(*GJB6*-D13S1830) [15], del(*GJB6*-D13S1854) [16] and del(131-kb) [17], respectively) are thought to eliminate a hypothesized cis-acting regulatory element located far upstream of *GJB2*. While the 920-kb and del(131-kb) deletions and the g. -77C>T promoter mutation seem to be private mutations, the del(*GJB6*-D13S1830) and del(*GJB6*-D13S1854) deletions and the c. -23+1G > A splice-site mutation are frequent in specific populations [16,18–22]. All of these mutations have been isolated in compound heterozygosity with *GJB2*-coding sequence mutations.

DFNB1 hearing impairment is clinically heterogeneous because of intrafamiliar and interfamiliar phenotypic variability, even in association with a same genotype [23]. The most common form is prelingual (onset before the acquisition of speech), non-progressive and severe or profound, affecting all frequencies. However, postlingual, progressive and moderate or mild hearing losses have also been reported, often associated with a few specific mutations [23]. Identification of these genotype-phenotype correlations is important to improve the accuracy of genetic counselling.

In this work, we studied a Spanish pedigree with three siblings affected by a mild, postlingual NSHI. We identified a novel *GJB2* mutation, which is the first one shown to alter the acceptor splice site of the single intron of the *GJB2* gene. Investigation of its effects on *GJB2* expression provided a likely explanation of the molecular mechanism underlying this mild DFNB1 phenotype.

## Materials and Methods

### Ethics statement

This study was approved by the Ethical Committee for Clinical Research of Hospital Universitario Ramón y Cajal. The study complied with the Spanish laws for biomedical research currently in force and adhered to the tenets of the Declaration of Helsinki.

### Subjects and clinical tests

Written informed consent was obtained from all the subjects included in the study. Syndromic features or putative environmental causes of HI were excluded in all affected subjects. HI was evaluated by pure-tone audiometry, testing for air conduction (frequencies 125–8,000 Hz) and for bone conduction (frequencies 250–4,000 Hz). The degree of HI was determined by calculating the binaural mean of the hearing thresholds for air conduction at frequencies 0.5, 1, and 2 kHz, and it was classified as mild (average thresholds in the range of 21–40 dB), moderate (41–70 dB), severe (71–90 dB), or profound (>90 dB). The HI of subject III:2 was also evaluated by auditory brainstem response (ABR) recording.

### DNA purification and assay procedures

DNA was extracted from peripheral blood samples by standard procedures. Genetic tests for mutations in the *GJB2* gene and for the large deletions affecting the *GJB6* gene at the DFNB1 locus were carried out as published [11,16]. Mutation nomenclature is based on cDNA sequence (GenBank accession number NM_004004.5) and follows current Human Genome Variation Society rules as implemented by the Mutalyzer 2.0β program (http://mutalyzer.nl).

The restriction fragment length polymorphism (RFLP) assay for the c. -22-2A>C mutation was carried out by digesting a 907-bp PCR product containing *GJB2* exon 2 and the intron 1/exon 2 boundary (obtained with primers 5’-ACCTGTTTTGGTGAGGTTGTGT-3’ and 5’-TGATCACGGGTTGCCTCATC-3’) with restriction endonuclease *Eco*88I, as recommended (Fermentas). The mutation creates a unique *Eco*88I site. Digestion products were as follows: wild-type allele: 907 bp; c. -22-2A>C allele: 770 bp + 137 bp.

Analysis of splice sites in the *GJB2* intronic sequence was performed by NNSplice software, version 0.9 (http://www.fruitfly.org/seq_tools/splice.html) [24].

### RNA purification and assay procedures

Saliva samples were collected in Oragene RNA Self-Collection Vials (DNA Genotek) according to the instructions of the manufacturer and stored at -20^°^C. Total RNA was extracted from 1 mL saliva samples following the Oragene RNA Purification protocol (DNA Genotek) with an RNeasy Micro kit (Qiagen). The protocol includes a 15-min incubation step with RNase-free DNase I (Qiagen) to remove contaminating DNA. cDNA was synthesized with 500 ng total RNA as template and random hexamer primers by using Superscript II reverse transcriptase (Life Technologies) as recommended.

To identify *GJB2* splicing products, we amplified cDNA by PCR by using FastStart DNA polymerase with GC-Rich Solution (Roche) and the following procedure: template cDNA was denatured for 5 min at 95^°^C, followed by 40 cycles of denaturation at 94^°^C (30 sec), annealing at 60^°^C (30 sec) and extension at 72^°^C (30 sec), and a final extension step at 72^°^C for 7 min. *GJB2*-specific primers were designed in exon 1 (forward primer: 5’-CGCGCTCCTCTCCCCGACT-3’) and exon 2 (reverse primer: 5’-TCCTTTGCAGCCACAACGAGGAT-3’) to rule out amplification of any contaminating genomic DNA. The reverse primer was used either unlabelled or labelled with 6-FAM at the 5’ end. Labelled PCR products were treated with T4 DNA polymerase (Roche) to blunt any 3’ protruding termini and were subsequently resolved by capillary electrophoresis in an ABI Prism 3130 Genetic Analyzer (Life Technologies). Unlabelled PCR products were separated in a 1.5% SeaKem GTG agarose gel; bands were excised and DNA was recovered by using NucleoSpin Extract II (Macherey-Nagel). Gel-extracted PCR products were either directly sequenced or cloned in the T-vector pCR2.1-TOPO with the TOPO TA Cloning kit (Life Technologies) and subsequently sequenced.

Quantification of *GJB2* splicing products was performed by real-time quantitative PCR (RT-qPCR) in a 7300 Real Time PCR System (Life Technologies). Specific primer pairs were designed with Primer Express software (Life Technologies) for the following products: *GJB2* transcripts derived from the known acceptor splice-site (5’-TCCCGACGCAGAGCAAAC-3’ and 5’-TGCAGCGTGCCCCAAT-3’), *GJB2* transcripts derived from alternative splice site 1 (5’-TCCCGACGCAGCTAGTGAT-3’ and 5’-GCGGTTTGCTCTGGAAAAGA-3’) and control *GAPDH* transcripts (5’-GGTCGGAGTCAACGGATTTG-3’ and 5’-AAACCATGTAGTTGAGGTCAATGAAG-3’). For each assay, we analyzed different amounts of cDNA (25, 125 and 625 ng) in duplicate. RT-qPCR reactions were carried out with 8 pmol of each primer by using the SYBR Green qPCR kit (Takara Bio) with the following amplification procedure: incubation at 50^°^C (2 min); denaturation at 95^°^C (10 min); 60 cycles of denaturation at 95^°^C (15 sec), annealing at 59^°^C (30 sec) and extension at 72^°^C (30 sec); and a final dissociation step of 95^°^C (15 sec), 60^°^C (30 sec) and 95^°^C (15 sec). Fluorescence was measured once per cycle, at the end of the extension step. Analysis of the relative amounts of each *GJB2* splicing product as regards the expression of the reference gene *GAPDH* was performed by the method of Pfaffl [25]. Results are the means of at least three independent experiments. Statistical analysis of the difference between means of the expression levels of transcripts from normal-hearing controls and subjects was performed by a two-tailed, paired t test, as implemented by InStat 3.05 program (GraphPad Software).

## Results

### A novel splice-site mutation in GJB2

**Figure 1:**
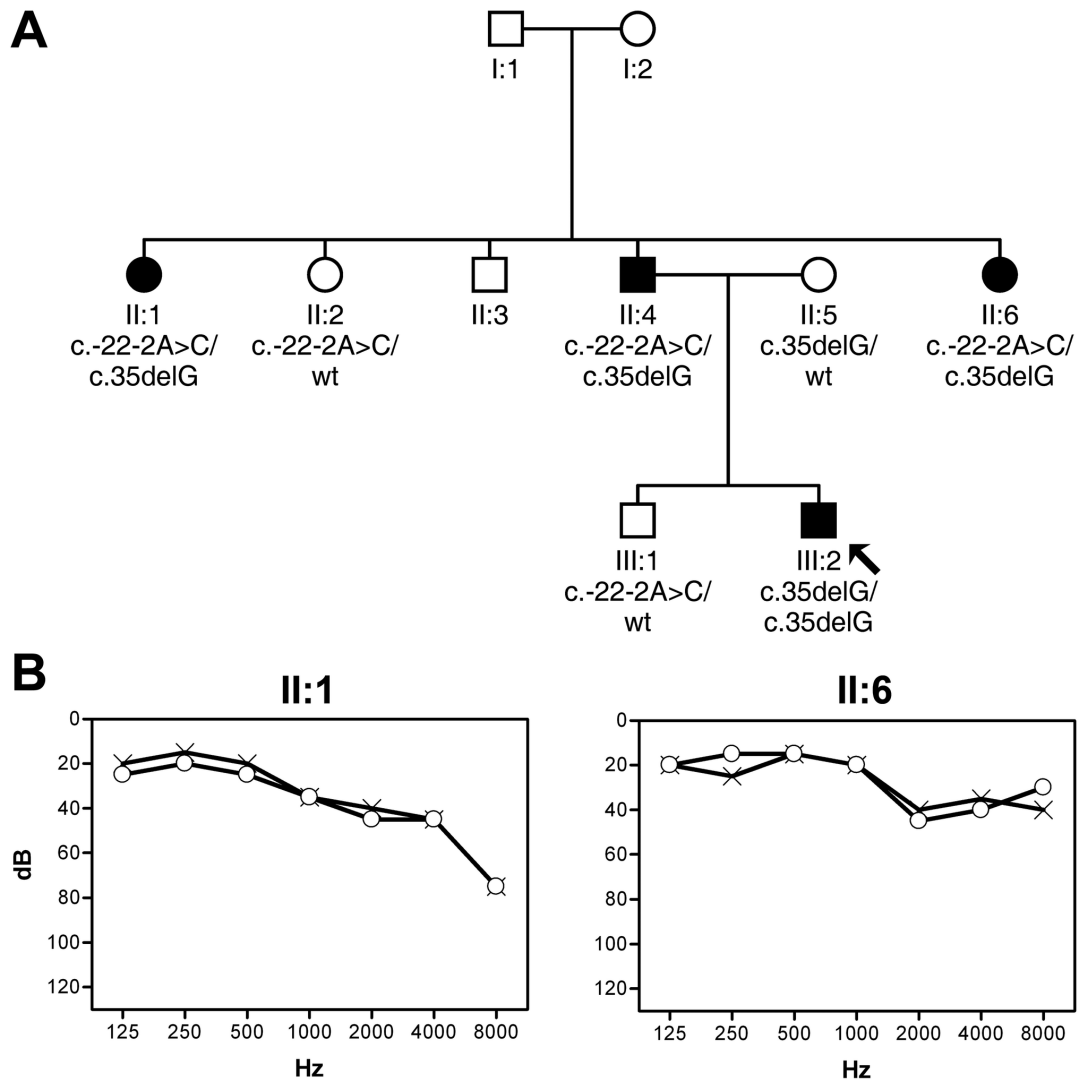
Hearing impairment in family S1599. **A** Pedigree of family S1599, indicating the *GJB2* genotypes of subjects who were analyzed. B Audiograms (air conduction) of affected individuals II:1 and II:6 at ages 50 and 31 years, respectively (circles, right ear; crosses, left ear). The binaural means of the hearing thresholds for air conduction at frequencies 0.5, 1, and 2 kHz for these subjects are 33.3 and 25.8 dB, respectively, corresponding to mild HI (21-40 dB).

We ascertained a family with nonsyndromic sensorineural HI that apparently segregated with an autosomal dominant inheritance pattern (family S1599; Figure 1A). Clinical presentation of the HI was heterogeneous: subject III:2, the index case, had severe prelingual deafness, whereas his father (II:4) and two paternal aunts (II:1 and II:6) had mild HI of postlingual onset (third-fourth decade of life; Figure 1B).

Molecular screening of the *GJB2* gene revealed that subject III:2 was homozygous for the frequent c.35delG *GJB2* mutation. The remaining affected subjects (II:4, II:1 and II:6) were compound heterozygous for c.35delG and a novel point mutation at the acceptor splice site of the single *GJB2* intron, c. -22-2A>C (IVS1-2A>C). Two normal-hearing subjects, II:2 and III:1 (paternal aunt and brother of the index case, respectively) were carriers of c. -22-2A>C. Therefore, this family segregated autosomal recessive DFNB1 HI with a pseudo-dominant pattern and presented intra-familial phenotypic variability. Interestingly, the phenotype of mild postlingual HI was associated with the c.35delG/c.-22-2A > C genotype.

We designed an *Eco*88I-RFLP assay to screen for the c. -22-2A>C mutation. The mutation was not found in 92 normal-hearing control individuals (184 chromosomes). It was not found either in over 1,500 unrelated Spanish subjects with HI from our laboratory collection. Examination of the databases from the 1000 Genomes Consortium (http://browser.1000genomes.org/index.html) [26] and NHLBI GO Exome Sequencing Project (http://evs.gs.washington.edu/EVS/) [27] indicated that the c. -22-2A>C variant (rs201895089) has been identified heterozygously in Caucasian individuals with a very low frequency (7 out of 10,778 alleles, or 0.00065).

Given the late onset of HI in affected individuals with c. -22-2A>C, we screened 13 unrelated subjects from our laboratory collection who had late-onset HI (presbycusis). The mutation was not found in any of them.

### Functional analysis of the c. -22-2A>C mutation

**Figure 2:**
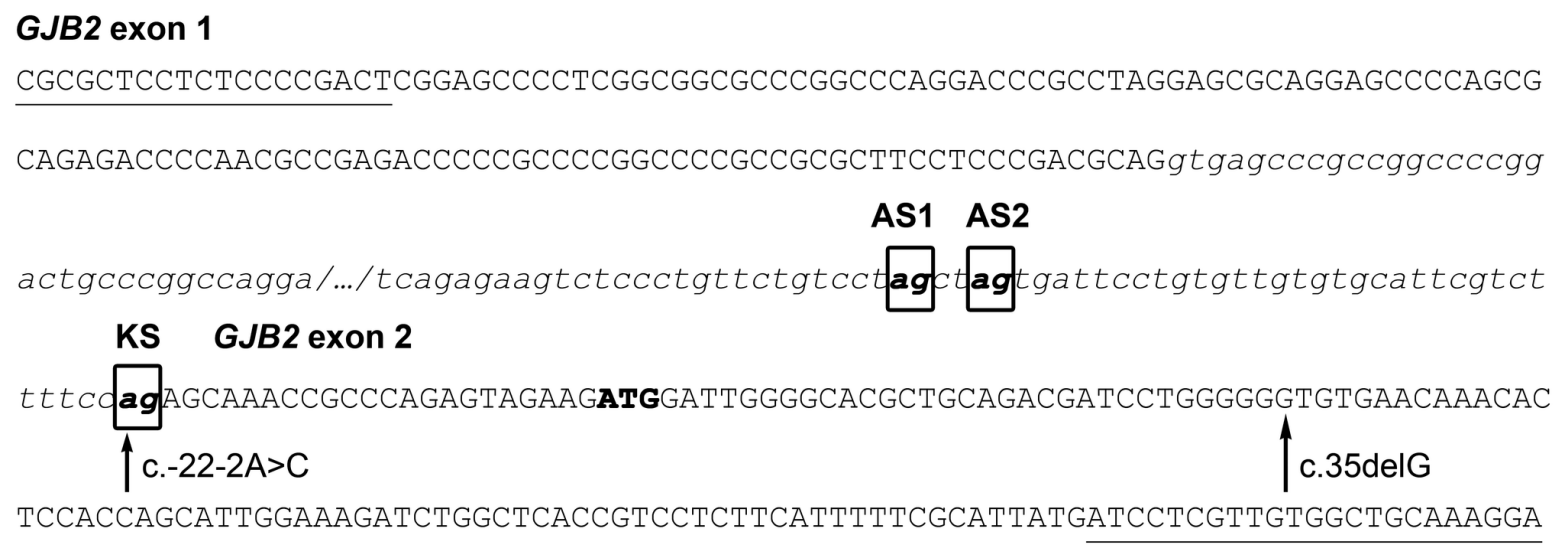
Sequence of the exon-intron junctions of the single *GJB2* intron. Exonic sequence is shown in capitals, whereas intronic sequence is shown in lowercase italics. The three acceptor splice sites identified in this work appear boxed in bold: KS, known acceptor site; AS1, alternative site 1; AS2, alternative site 2. The ATG start codon appears in bold. Nucleotides affected by the c. -22-2A>C and c.35delG mutations are indicated by arrows. Locations of the primers used for identifying *GJB2* splicing products are underlined.

Mutation c. -22-2A>C affected one of the two invariant nucleotides of the acceptor splice site of the *GJB2* intron. Accordingly, *in silico* analysis of the mutant splice-site with NNSplice software predicted that the site would be abolished. However, NNSplice also predicted the existence of an alternative acceptor splice site located 38 bp upstream of the previously known acceptor site (Figure 2); both sites had similar, high-quality scores (0.88 for the known acceptor site and 0.82 for the alternative site, within a 0-to-1 probability scale). Use of this previously unrecognized acceptor splice site would result in the synthesis from the c. -22-2A>C allele of slightly longer *GJB2* transcripts, with 38 bp of intronic sequence inserted in the 5’ UTR, but otherwise encoding wild-type Cx26.

In the course of this work, we verified that RNA obtained from saliva samples was a source of *GJB2* transcripts. We amplified these transcripts by reverse-transcriptase PCR techniques to assay for the expression of *GJB2*. Forward and reverse PCR primers were located in exons 1 and 2, respectively. The amplification product included the site of the c.35delG mutation, in order to distinguish the c. -22-2A>C allele from the c.35delG one (which would be 1-bp shorter).

**Figure 3:**
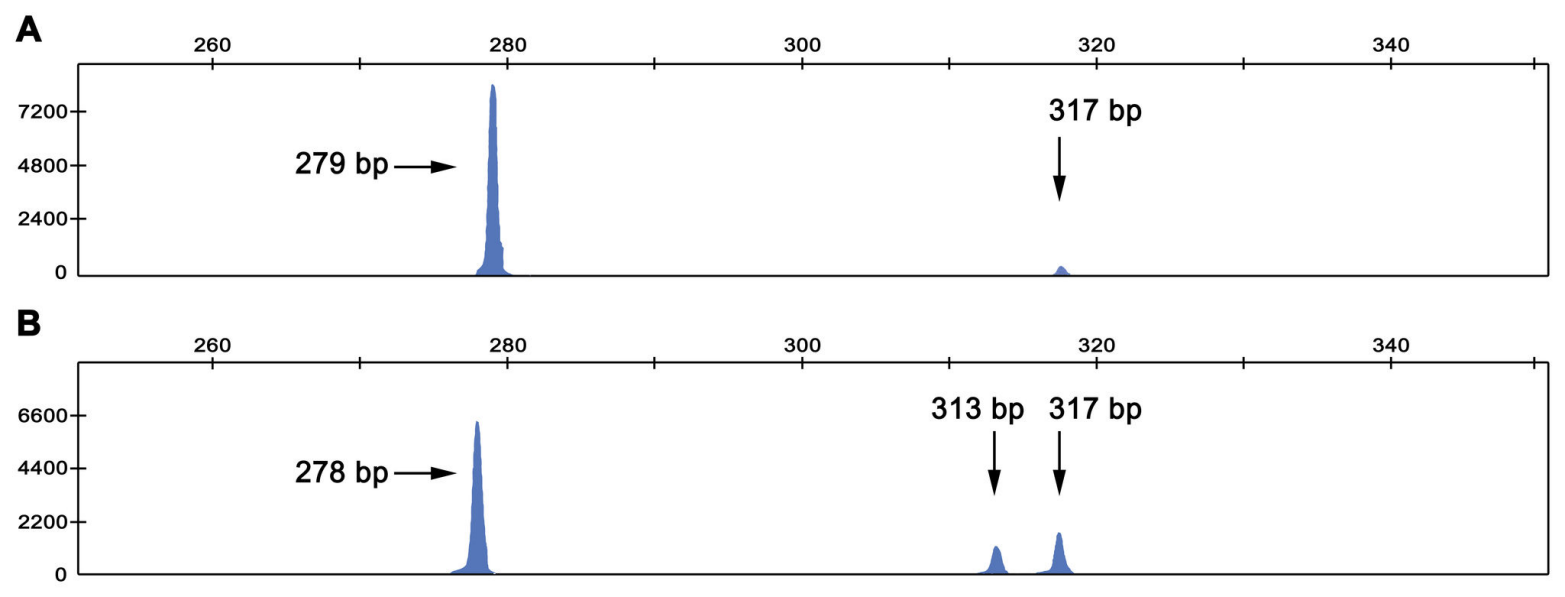
Analysis of *GJB2* splicing products. Splicing products from transcription of *GJB2* were amplified in a fluorescent RT-PCR assay performed with total RNA extracted from saliva samples and were separated by capillary electrophoresis. **A** *GJB2* splicing products from a normal-hearing control (genotype wt/wt). **B** *GJB2* splicing products from c. -22-2A>C/c.35delG compound heterozygous subject II:4.

For the assay, the reverse primer was labelled with a fluorophore, and blunted PCR products were resolved in a capillary fragment analyzer. We expected a 279-bp product if the known splice site was used and a 317-bp product in case that the alternative splice site predicted by NNSplice (hereafter referred to as “alternative site 1”) was used. In normal-hearing control individuals (genotype wt/wt), we observed two labelled PCR products at 279 and 317 bp (Figure 3A), suggesting that the two splice sites were indeed used, albeit with different efficiencies, as indicated by the much larger peak corresponding to use of the known splice site. In contrast, in the c.35delG/c.-22-2A > C compound heterozygote II:4, we observed three labelled PCR products of 278, 313 and 317 bp (Figure 3B). The absence of a 279-bp product confirmed that the known acceptor splice site is abolished in the c. -22-2A>C allele (the 278-bp product corresponds to the c.35delG allele). Also, as no 316-bp product was detected, the alternative site 1 from the 35delG allele seems to be minimally used or not used at all. The 313-bp product suggested the existence of a second alternative acceptor site (see below). Overall, in subject II:4, the amount of transcripts originating from the c. -22-2A>C allele was markedly inferior to that of transcripts from the c.35delG allele (Figure 3B).

**Figure 4:**
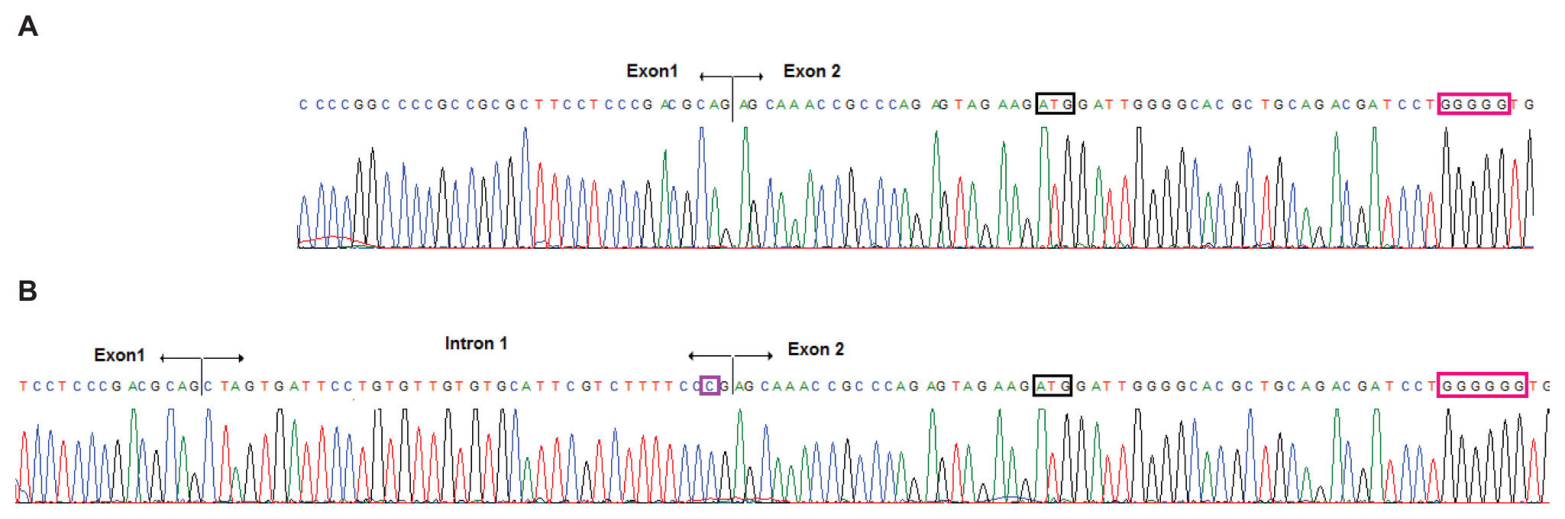
Sequence electropherograms of cloned *GJB2* transcripts from c. **-22-2A>C/c.35delG compound heterozygous subject II:4**. The stretch of six guanine nucleotides affected by the c.35delG deletion appears boxed in red, while the start ATG codon is boxed in black. **A** Sequence of the transcript derived from the c.35delG allele, generated by use of the known acceptor site. Note that the stretch of guanine nucleotides contains only five guanines. **B** Sequence of the transcript derived from the c. -22-2A>C allele generated by use of the alternative acceptor site 1. Note the cytosine nucleotide at c. -22-2, boxed in purple.

To verify our interpretation of these results, we repeated the assay with the reverse PCR primer unlabelled. PCR products were resolved in an agarose gel: in controls we observed two bands (that corresponded to the 279 and 317 bp products), while in the c.35delG/c.-22-2A > C subject we observed three bands (278, 313, and 317 bp). Bands were excised from the gel and purified (the 313 and 317 bp bands were purified together as it was not possible to excise them separately because of their proximity in the gel). Purified PCR products were directly sequenced; in parallel, the products were cloned in a T-vector and individual clones were sequenced. Sequencing showed that the 278/279 bp and 317 bp fragments did correspond to the transcripts generated by use of the known site and the alternative site 1, respectively (Figure 4). The 313-bp product was generated by use of a second alternative acceptor site (hereafter referred to as “alternative site 2”), located 4 bp downtream of the alternative site 1. Use of the alternative site 1 added 38 intronic nucleotides to the transcript, while use of the alternative site 2 just added 34 intronic nucleotides (Figure 2). The alternative site 2 is less used than site 1, as indicated by the assay performed in the capillary fragment analyzer (Figure 3B).

**Figure 5:**
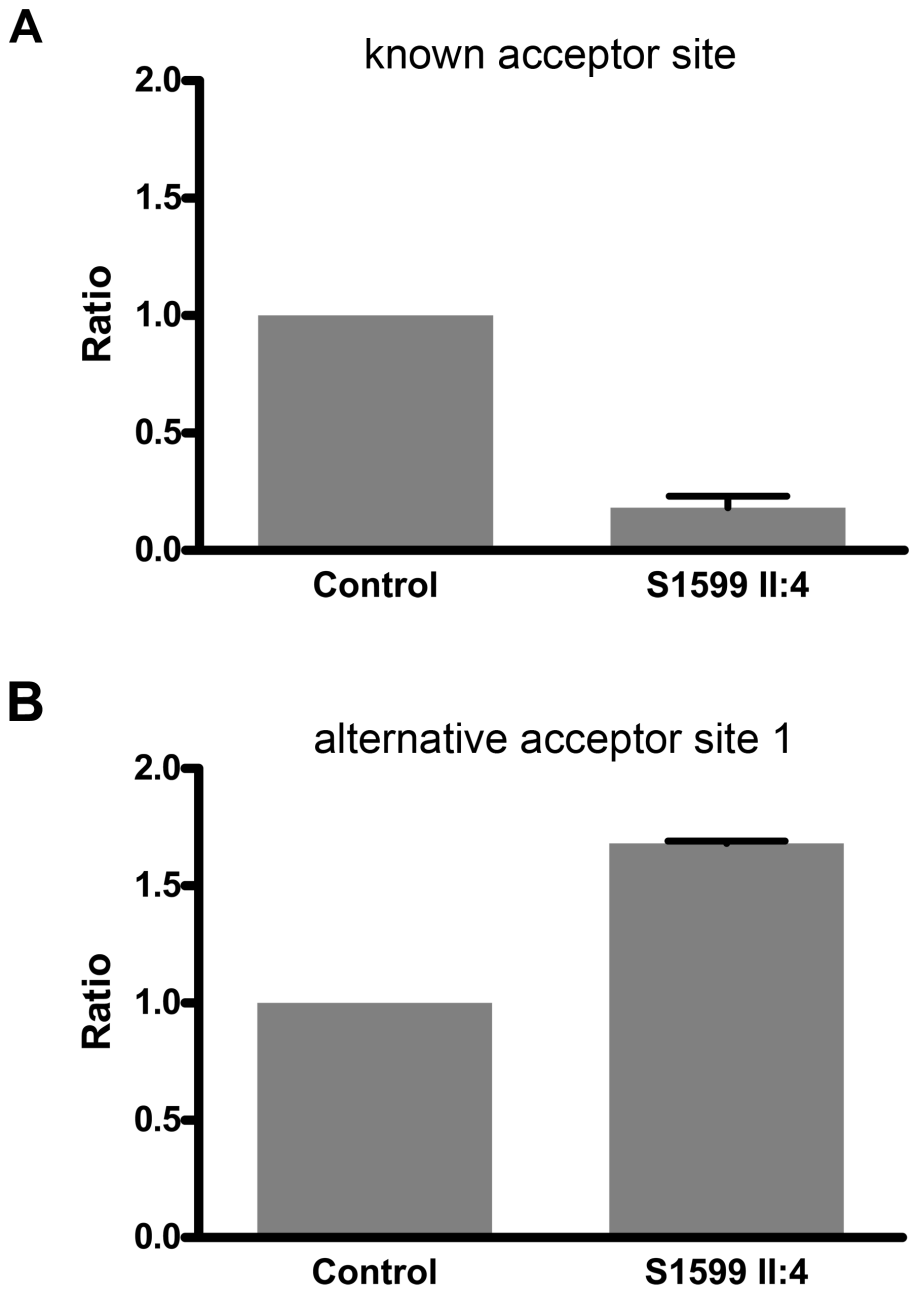
Relative expression levels of *GJB2* transcripts generated by splicing at different acceptor sites. Each panel shows results for a normal-hearing control (genotype wt/wt) and for c. -22-2A>C/c.35delG compound heterozygous subject II:4. **A** Relative expression of *GJB2* transcript generated by splicing from the known site (two-tailed *p* < 0.0001, N=5). B Relative expression of *GJB2* transcript generated by splicing from alternative site 1 (two-tailed *p*= 0.0094, N=3).

We quantified the relative amounts of *GJB2* cDNAs from subject II:4 by means of real-time qPCR. Data were normalized by comparing with data obtained from a control subject and referred to the levels of a ubiquitously expressed control gene (*GAPDH*). For these assays, primers were designed for the specific amplification of cDNAs obtained by splicing from the known site and the alternative site 1. The ratios for each transcript between II:4 and the wild-type control were 0.18 (known site) and 1.68 (alternative site 1) (Figure 5). Since the c. -22-2A>C mutation abolishes the known acceptor site, we expected that the amount of transcript from this site in subject II:4 (generated from the c.35delG allele) was about 50% that of the control. However, it is just 18%, which suggests that transcripts with c.35delG may be either less stable or less efficiently spliced than wild-type transcripts. In addition, our results indicate that abolition of the known site results in an increased use of alternative site 1.

## Discussion

We have identified a novel *GJB2* splice-site mutation (c. -22-2A>C) which belongs to the small group of DFNB1-causing mutations that lie outside the coding region of the *GJB2* gene. Only another pathogenic splice-site mutation, c. -23+1G > A [12], had been identified to date in this simple gene containing just two exons. The c. -23+1G > A and c. -22-2A>C mutations abolish the donor and acceptor splice sites of *GJB2* intron 1, respectively. Unlike many splice-site mutations, they have no direct effect on protein coding because the coding sequence of *GJB2* is completely contained within exon 2. Rather, their pathogenic effect is due to impaired expression of the mutant alleles. As regards c. -23+1G>A, *GJB2* transcripts generated from this allele were not detected in a lymphoblastoid cell line derived from a c. -23+1G>A/c.35delG compound heterozygote [28], suggesting that either the c. -23+1G > A allele was not transcribed or the transcript was unstable. This result agreed with the profound HI of this subject. Indeed, the c. -23+1G > A mutation is nearly always associated with severe or profound HI of prelingual onset when in homozygosity or in compound heterozygosity with truncating mutations such as c.35delG, c.167delT, c.235delC and c.290dupA [22,29–32]; it was reported in association with moderate HI only once, in one compound heterozygote for c. -23+1G > A and c.35delG [30]. By contrast, we did detect transcripts encoding wild-type Cx26 from the c. -22-2A>C allele of a c. -22-2A>C/c.35delG heterozygote, although in a much lesser amount than that of transcripts from the c.35delG allele. Although this residual expression does not suffice to maintain a normal phenotype, it is likely that synthesis of functional Cx26 from transcripts derived from the c. -22-2A>C allele may underlie the delayed onset and milder hearing phenotype observed in the three c. -22-2A>C/c.35delG heterozygotes of family S1599.

The degree of hearing loss associated with DFNB1 HI is very variable, ranging from mild to profound; this variability is apparent even in subjects with the same genotype, as shown in the large multicenter study of Snoeckx et al. [23]. This study also identified a small number of missense mutations (p.Met34Thr, p.Val37Ile and p.Leu90Pro) that were consistently associated with mild or moderate HI of postlingual onset. The c. -22-2A>C mutation seems to belong to this group of hypomorphic alleles given the hearing phenotype observed in the three affected siblings of family S1599 (mild HI of late postlingual onset). This correlation ought to be regarded as provisional, as it has been deduced from just three siblings. Yet, the lack of phenotypic variability among the affected siblings and the observed effects of c. -22-2A>C on *GJB2* expression support this conclusion.

The c. -23+1G > A mutation is the most frequent DFNB1 pathogenic allele in Mongolia [22] and a relatively frequent allele in the Kurdish, Turkish, Czech, Polish and Chinese populations [19–21,32,33]. On the contrary, c. -22-2A>C seems to be a relatively rare allele. Our screening procedure does not miss this mutation because we sequence both *GJB2* exons and exon-intron junctions in all cases in which DFNB1 HI is suspected. However, c. -22-2A>C is first reported here, and it has only been found once in our large cohort of HI cases from the Spanish population, which is consistent with its low allelic frequency (0.00065) in Caucasian populations, as reported by the 1000 Genomes and NHLBI Exome Sequencing projects. The fact that it has not been found in any other cohorts of subjects with HI studied so far might be just the consequence of an enrolment bias towards subjects with severe or profound HI, which would under-represent hypomorphic alleles associated with late-onset mild-to-moderate HI. Although we did not find the mutation in a few cases with presbycusis, a hypothetical involvement of c. -22-2A>C in this pathology deserves further exploration.
