# Correction: A Novel Splice-Site Mutation in the *GJB2* Gene Causing Mild Postlingual Hearing Impairment

**DOI:** 10.1371/annotation/571fea42-45d0-4dcc-a676-6709c60c2cad

**Published:** 2014-01-06

**Authors:** Marta Gandía, Francisco J. del Castillo, Francisco J. Rodríguez-Álvarez, Gema Garrido, Manuela Villamar, Manuela Calderón, Miguel A. Moreno-Pelayo, Felipe Moreno, Ignacio del Castillo

Several errors were introduced in the preparation of this article for publication.

The word "connexin" has been incorrectly substituted by "connection" throughout the article. 

